# Acute percheron infarction: a precision learning

**DOI:** 10.1186/s12883-022-02735-w

**Published:** 2022-06-04

**Authors:** Bei Zhang, Xiaoxun Wang, Chen Gang, Jiping Wang

**Affiliations:** 1 Department of Radiology, First Hospital of Jilin University, No. 1, Xinmin Street, Changchun, 130021 Jilin Province China; 2 Department of Radiology, Beijing Canaan clinic Co., Ltd, Beijing, China; 3 Department of Radiology, Binzhou Medical University Hospital, Binzhou, China

**Keywords:** Cerebral Infarction, Thalamus, Diagnostic imaging

## Abstract

**Background:**

So far, the diagnosis of acute artery of percheron (AOP) infarction is uncommon. In this study, patients with acute AOP infarction were studied to explore the relationship of imaging findings, clinical manifestations and prognosis of acute AOP infarction.

**Materials:**

A total of 23 patients with acute AOP infarction in our institution from 2014 to 2019 were reviewed retrospectively. All cases were evaluated by computed tomography (CT) and magnetic resonance imaging (MRI). The modified Rankin scale (MRS), blood examination, electrocardiogram and transthoracic echocardiography were used for detailed clinical and prognostic evaluation. All standard risk factors for these patients were recorded. The MRS scores were performed 90 days after discharge.

**Results:**

Four different types of acute AOP infarction were identified: (a) bilateral paramedian thalamic infarction (BPTI, 52%); (b) bilateral paramedian thalamic with rostral midbrain infarction (BPTRMI, 30%), (c) bilateral paramedian and anterior thalamic infarction (BPATI, 13%), and (d) bilateral paramedian thalamic with red nuclei infarction (BPTRNI, 4%). These patients had consciousness disorder, memory dysfunctions, vertical gaze paresis and mesencephalothalamic syndrome. The 65% of patients with BPTI and BPATI experienced relatively good functional recovery and could carry out daily life activities (MRS score ≤ 2). However, patients with BPTRMI may have an unfavorable outcome.

**Conclusions:**

Although the clinical features are variable, DWI or ADC map can improve the diagnosis of acute AOP infarction patterns. Acute AOP occlusion requires immediate diagnosis and treatment to obtain more favorable outcome and avoid additional unnecessary procedures.

## Introduction

**Fig. 1:**
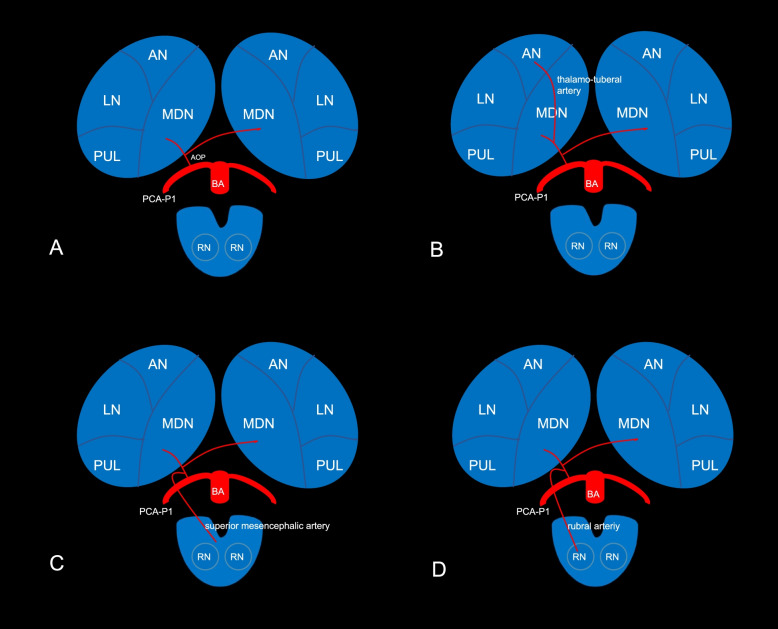
Different anatomic regions of the thalamus and its arterial supply. AOP is a solitary arterial trunk of bilateral thalamo-perforating artery originated from P1 segment of one of PCA, which supplies bilaterally the paramedian thalamic territories (**A**). When the polar artery is absent, AOP not only may supply the paramedian but also the anterior thalamic territories (**B**). The rostral midbrain has supplied by the superior mesencephalic artery, which share a common origin with AOP (**C**). The red nucleus has supplied by the rubral artery, which share a common origin with AOP (**D**). AOP, artery of percheron; PCA, posterior choroidal artery; BA, basilar artery; AN, anterior nucleus; LN, lateral nuclei; MDN, mediodorsal nuclei; PUL, pulvinar; RN, red nucleus

The arterial supply of thalamus is divided into four regions: the anterior part is supplied by polar artery or thalamic tubercle artery; the paramedian is supplied by the paramedian artery or thalamic perforating artery; the inferolateral supplied by the thalamo-geniculate artery, and the posterior supplied by the posterior choroidal artery [1–3]. Perceron G [4] first described the artery of percheron (AOP) in 1973. It is a solitary arterial trunk of the bilateral thalamo-perforating artery originating from P1 segment of the posterior cerebral artery (PCA), which supplies bilaterally the paramedian thalamic territories. The AOP is an uncommon arterial variant. Meanwhile, rostral midbrain has supplied by the superior mesencephalic or rubral arteries, which can branch separately from P1 segment of the PCA or share a common origin with AOP (Fig. 1). Thus, AOP probably supply the rostral midbrain or red nucleus [5–7].

Acute AOP infarction is a rare pattern of ischemia. It can significantly affect patients’ health and may be life-threatening, although the ischemic lesion of paramedian bichulami is relatively small [5, 8]. Therefore, the diagnosis of acute AOP infarction is very important to guide appropriate treatment and prevent additional unnecessary disposal measures [8].

The diameter of AOP is so small that magnetic resonance angiography (MRA) cannot show it, and even digital subtraction angiography (DSA) cannot show its existence, stenosis or occlusion in most cases. Only a few authors found AOP obstruction through DSA [9, 10]. MRI, especially diffusion weighted imaging (DWI), plays an important role in the diagnosis of acute AOP infarction. At present, most neuroimaging literatures only describe the relationship between clinical manifestations and imaging [11, 12]. However, in acute AOP infarction cases, whether imaging findings can suggest prognosis needs to be further confirmed. Whether there is a certain correlation between the clinical characteristics and prognosis of different types of acute AOP infarction information remains to be summarized.

## Materials and methods

The study was approved by the institutional review committee. We retrospectively identified 23 patients with acute AOP infarction by searching the medical chart database from 2012 to 2021 (key words: bilateral acute paramedian thalamic infarction).

Inclusion criteria: 1) The clinical manifestations of acute AOP infarction can be found in the medical chart database; 2) All the patients underwent MRI, including axial T2-weighted images (T2WI), axial T1-weighted images (T1WI), axial fluid-attenuated inversion-recovery (FLAIR) and DWI sequence; 3) High signal intensity on DWI or low signal intensity on apparent diffusion coefficients (ADC) map were noted in bilateral paramedian thalami with or without rostral midbrain involvement; 4) Neurological examination was performed during hospitalization; 5) Three months after discharge, modified Rankin Scale (MRS) score was performed; and 6) All the patients were followed up for at least 6 months.

**Fig. 2:**
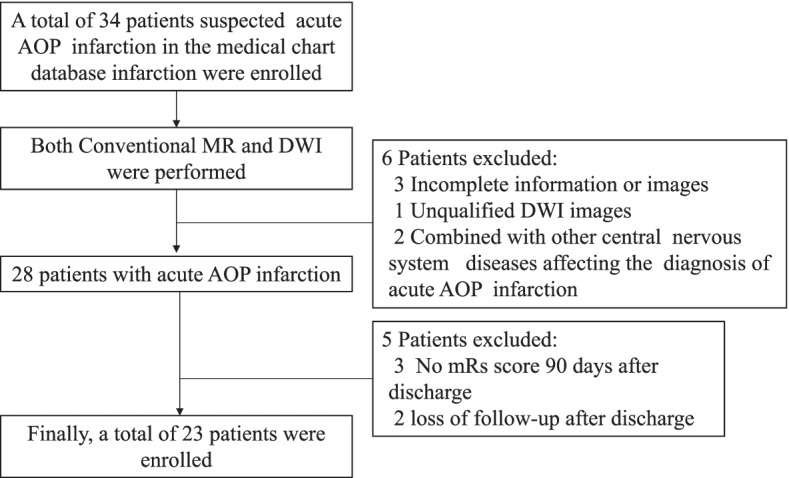
Steps in consideration of individual data for inclusion

Exclusion criteria: 1) Patients with the top of the basilar artery syndrome, diffuse midline glioma of thalamus, viral encephalitis, arteriovenous fistula (AF) and wernicke encephalopathy; and 2) Patients with incomplete examination or medical records. Steps in consideration of individual data for inclusion were shown in Fig. 2.

### Clinical data

Clinical workup included assessment of cardiovascular risk factors, diabetes mellitus history and evaluation of coagulation function. Evaluate the clinical risk factors of each patient, including cardiac embolism, large artery atherosclerosis or small artery occlusion.

### Image analysis

Images of all patients with acute AOP infarction were reviewed by 2 senior neuroradiologists. In the Kappa consistency test of the 2 radiologists, Kappa value = 0.750, *p* <0.001. The acute ischemic territories involved in each patient were recorded under the following categories: only paramedian bithalamus (asymmetric or symmetric), paramedian bithalamic with rostral midbrain, paramedian bithalamic with anterior thalamus (right, left or bilateral), paramedian bithalamus and anterior thalamic with rostral midbrain and paramedian bithalamic with red nucleus (right, left or bilateral). Acute infarction was defined as hyperintense in DWI and hypointense in ADC map.

## Results

### Clinical syndromes

Table 1 shows the risk factors and neurological symptoms for all cases (age range, 23 to 86 years; mean, 58 ± 14 years; 8 women, 15 men). The most common syndrome was altered mental status in 22 cases (96%), which include 11 cases of hypersomnia (48%), 7 cases of coma (30%) and 4 cases of drowsiness (17%). Other characteristic included: vertical gaze palsy in 8 cases (35%), hypomnesis in 7 cases (30%), oculomotor nerve palsy in 7 cases (30%), disorientation in 5 cases (22%), barylalia and aphasia in 6 cases (26%), movement disorders in 9 cases (39%), ataxia in 5 cases (22%) and hemiplegia in 2 cases (9%).

**Table 1:** Clinical and demographic characteristics of the whole cohort at baseline visit

Patient No./ Age(y)/Gender	Admission time since onset of symptoms	Subgroups of acute AOP infarction	Risk factors	Aetiologies	Symptoms	MRS after 90 days	Treatment
1/65/F	1 hour	Paramedian bithalami	Cho, DM, HTN	Arteriosclerosis of right PCA	Hypersomnia; Vertical gaze palsy.	2	Aspirin 200 mg/d orally
2/71/F	2 hours	Paramedian bithalami rostral midbrain	Smoke, DM, HTN, CAD	Small vessel disease	Coma; Vertical gaze palsy; Oculomotor nerve palsy; Movement disorders.	3	Aspirin 200 mg/d orally
3/68/M	3 hours	Paramedian bithalami	Smoke, Cho, DM, HTN, CAD	Arteriosclerosis of BA	Hypersomnia; Movement disorders; Aphasia; Hypomnesis.	2	Aspirin 200 mg/d combined with clopidogrel 7.5 mg/d
4/66/M	1 hours	Paramedian bithalami	DM, HTN, CAD	Arteriosclerosis of left PCA	Coma; Movement disorders.	0	Alteplase
5/63/M	2 hours	Paramedian bithalami anterolateral thalami	Smoke, Drinking, HTN	Sinus bradycardia	Hypersomnia; Hypomnesis; Disorientation; Movement disorders; Corticospinal tract signs.	2	Low-molecular-weight heparins calcium injection (4000 IU/12 h)
6/69/M	5.5 hours	Paramedian bithalami rostral midbrain	Cho, DM, HTN, CAD	Arteriosclerosis of left PCA	Hypersomnia; Oculomotor nerve palsy; Movement disorders.	4	Aspirin 200 mg/d orally
7/45/M	8 hours	Paramedian bithalami red nucleus	Cho, DM, HTN	Atrial fibrillation	Hypersomnia; Barylalia; Ataxia; Dysarthria.	3	Low-molecular-weight heparins calcium injection (4000 IU/12 h)
8/64/M	1 hours	Paramedian bithalami	Smoking, Cho, DM, HTN	Small vessel disease	Hypersomnia; Barylalia; Movement disorders.	1	Alteplase
9/60/M	2.5 hours	Paramedian bithalami rostral midbrain	Cho, DM, HTN	Atrial fibrillation	Hypersomnia; Disorientation; Hypomnesis; Movement disorders.	3	Low-molecular-weight heparins calcium injection (4000 IU/12 h)
10/67/M	10.5 hours	Paramedian bithalami rostral midbrain	Smoke, Cho, DM, HTN	Small vessel disease	Coma; Vertical gaze palsy; Hypomnesis; Disorientation; Oculomotor nerve palsy; Movement disorders.	4	Aspirin 200 mg/d combined with clopidogrel 7.5 mg/d
11/29/F	4.5 hours	Paramedian bithalami anterolateral thalami	Smoke	Small vessel disease	Coma; Hypomnesis; Disorientation.	1	Alteplase
12/43/F	2.5 hours	Paramedian bithalami	Cho, DM, HTN	Endocarditis	Coma; Hemiplegia.	1	Low-molecular-weight heparins calcium injection (4000 IU/12 h)
13/77/M	6 hours	Paramedian bithalami rostral midbrain	Smoking, DM, HTN	Atrial fibrillation	Coma; Vertical gaze palsy; Disorientation; Oculomotor nerve palsy; Movement disorders.	4	Low-molecular-weight heparins calcium injection (4000 IU/12 h)
14/55/F	5.5 hours	Paramedian bithalami	Cho, DM, HTN	Small vessel disease	Cerebellar ataxia; Barylalia.	2	Alteplase
15/60/M	1 hour	Paramedian bithalami	Smoking, Cho, HTN	Small vessel disease	Drowsiness; Vertical gaze palsy.	2	Aspirin 200 mg/d orally
16/67/M	6 hours	Paramedian bithalami	Cho, DM, HTN	Small vessel disease	Hypersomnia; Barylalia.	2	Alteplase
17/53/M	1.5 hours	Paramedian bithalami	Smoke	Atrial myxoma	Drowsiness; Movement disorders.	1	Low-molecular-weight heparins calcium injection (4000 IU/12 h)
18/64/F	2 hours	Paramedian bithalami rostral midbrain	Cho, DM, HTN	Arteriosclerosis of right PCA	Drowsiness; Cerebellar ataxia; Vertical gaze palsy; Oculomotor nerve palsy; Movement disorders.	4	Aspirin 200 mg/d combined with clopidogrel 7.5 mg/d
19/66/M	2.5 hours	Paramedian bithalami anterolateral thalami	Cho, DM	Small vessel disease	Hypersomnia; Hypomnesis; Vertical gaze palsy.	1	Alteplase
20/75/F	3 hours	Paramedian bithalami rostral midbrain	Cho, DM, HTN	Atrial myxoma	Drowsiness; Cerebellar ataxia; Vertical gaze palsy; Oculomotor nerve palsy; Movement disorders.	5	Aspirin 200 mg/d combined with clopidogrel 7.5 mg/d
21/23/M	4 hours	Paramedian bithalami	Smoke	Basilar artery dissection	Drowsiness.	2	Aspirin 200 mg/d combined with clopidogrel 7.5 mg/d
22/30/F	3 hours	Paramedian bithalami	–	Basilar artery dissection	Coma; Aphasia; Movement disorders.	2	Aspirin 200 mg/d combined with clopidogrel 7.5 mg/d
23/65/M	1 hour	Paramedian bithalami	Smoking, Drinking,Cho,DM	Small vessel disease	Hypersomnia; Hypomnesis; Hemiplegia.	1	Aspirin 200 mg/d combined with clopidogrel 7.5 mg/d

*Abbreviations*: *M* male, *F* female, *Cho* hypercholesterolemia, *DM* diabetes mellitus, *HTN* arterial hypertension, *PCA* posterior cerebral artery, *BA* basilar artery, *AF* atrial fibrillation, *CAD* coronary artery disease

### Imaging findings

**Fig. 3:**
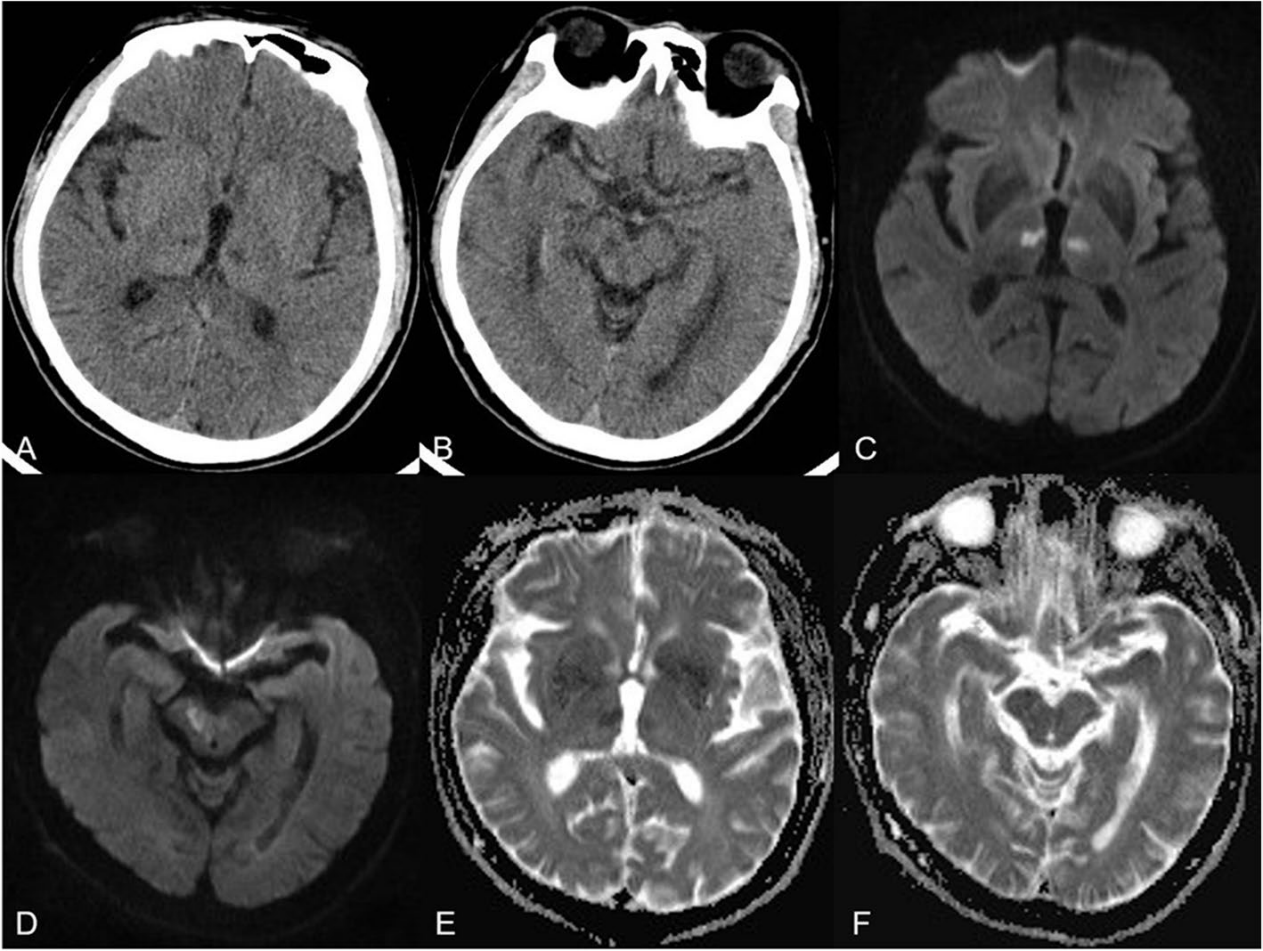
Bilateral parathalamic and rostral midbrain hypodensity on axial head CT. Case 2. Axial head CT at 2 hours after onset (**A** and **B**) shows hypodensity in the bilateral paramedian thalamus and midbrain involvement (white arrow). Axial DWI and ADC maps (**C** to **F**) show acute symmetrical infarctions in the bilateral paramedian thalamus and show a V-shaped acute infarction at the interpeduncular fossa of midbrain

**Fig. 4:**
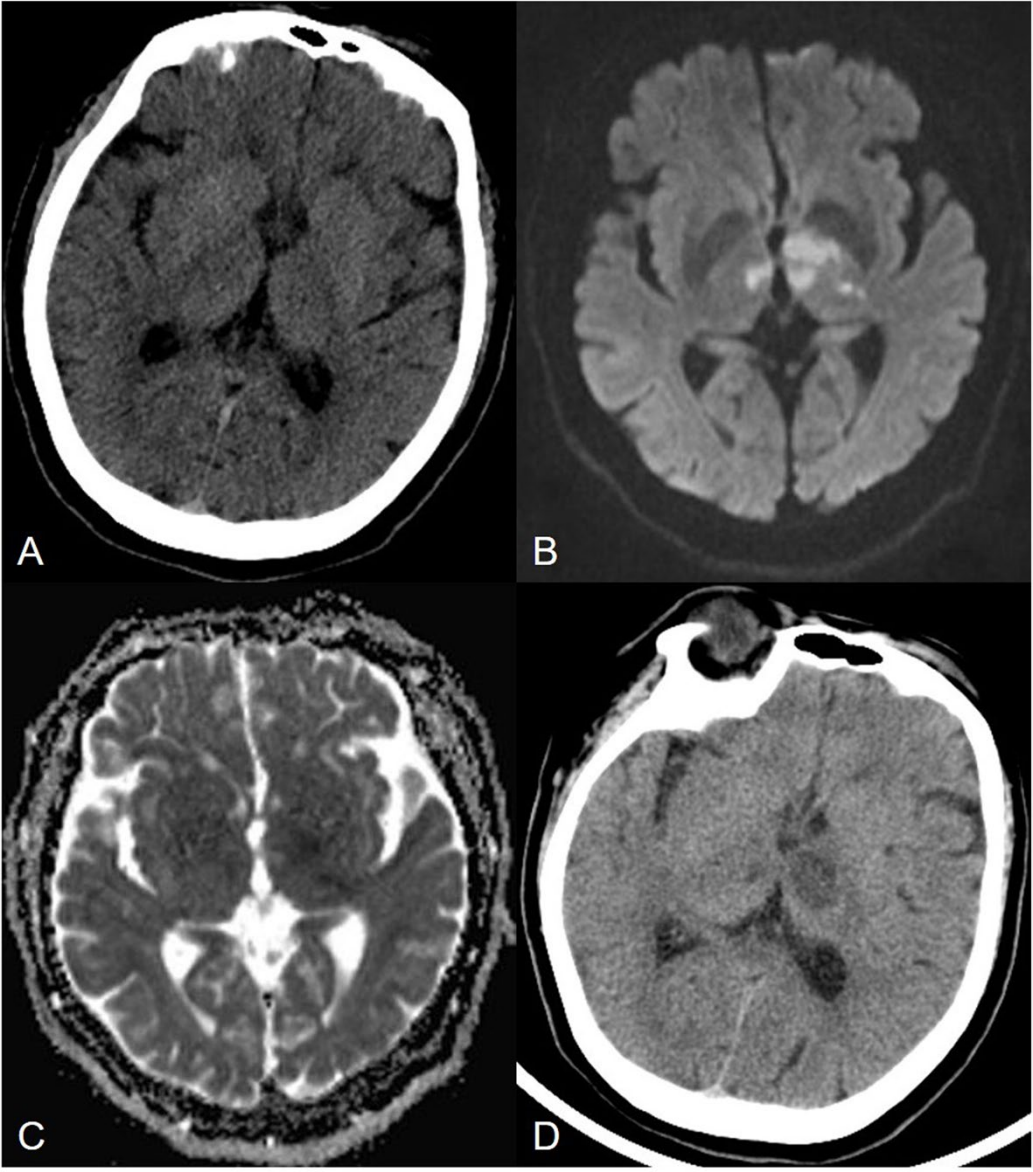
Bilateral paramedian thalamic ischemia with anterolateral paramedian territory. Case 5. Axial head CT at 2 hours after onset (**A**) shows no hypodensity. Axial DWI and ADC map show acute infarcts (**B** and **C**) in the bilateral paramedian thalami (white arrow) and left anterior thalami (black arrow) after 24 hours, meanwhile, head CT show hypodensity (**D**)

**Fig. 5:**
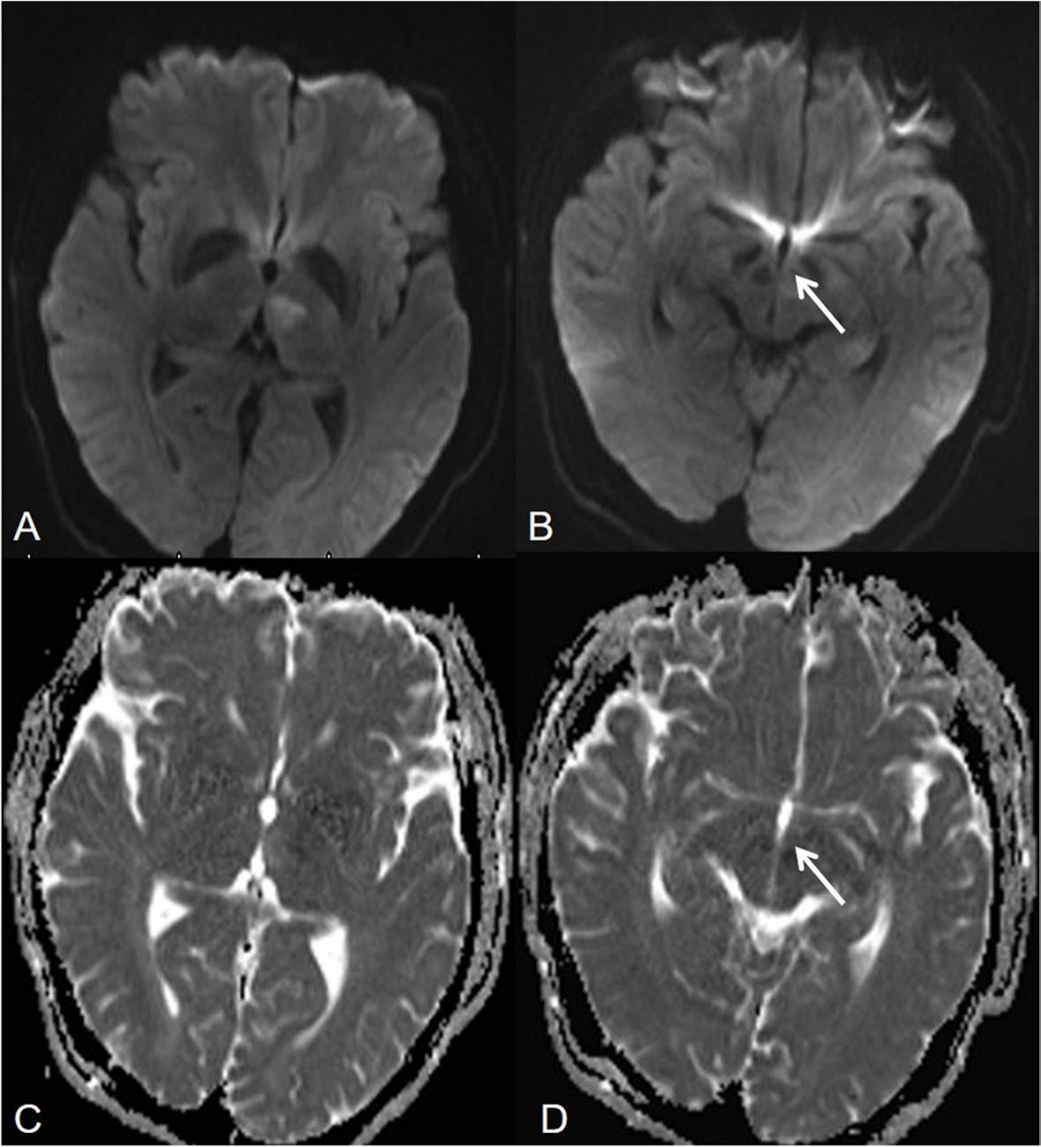
Bilateral paramedian thalamic infarction with left red nucleus. Case 7. Axial DWI and ADC map demonstrate acute symmetrical infarcts in the bilateral paramedian thalami (**A**, black arrow) and left red nucleus (**B**, white arrow)

**Fig. 6:**
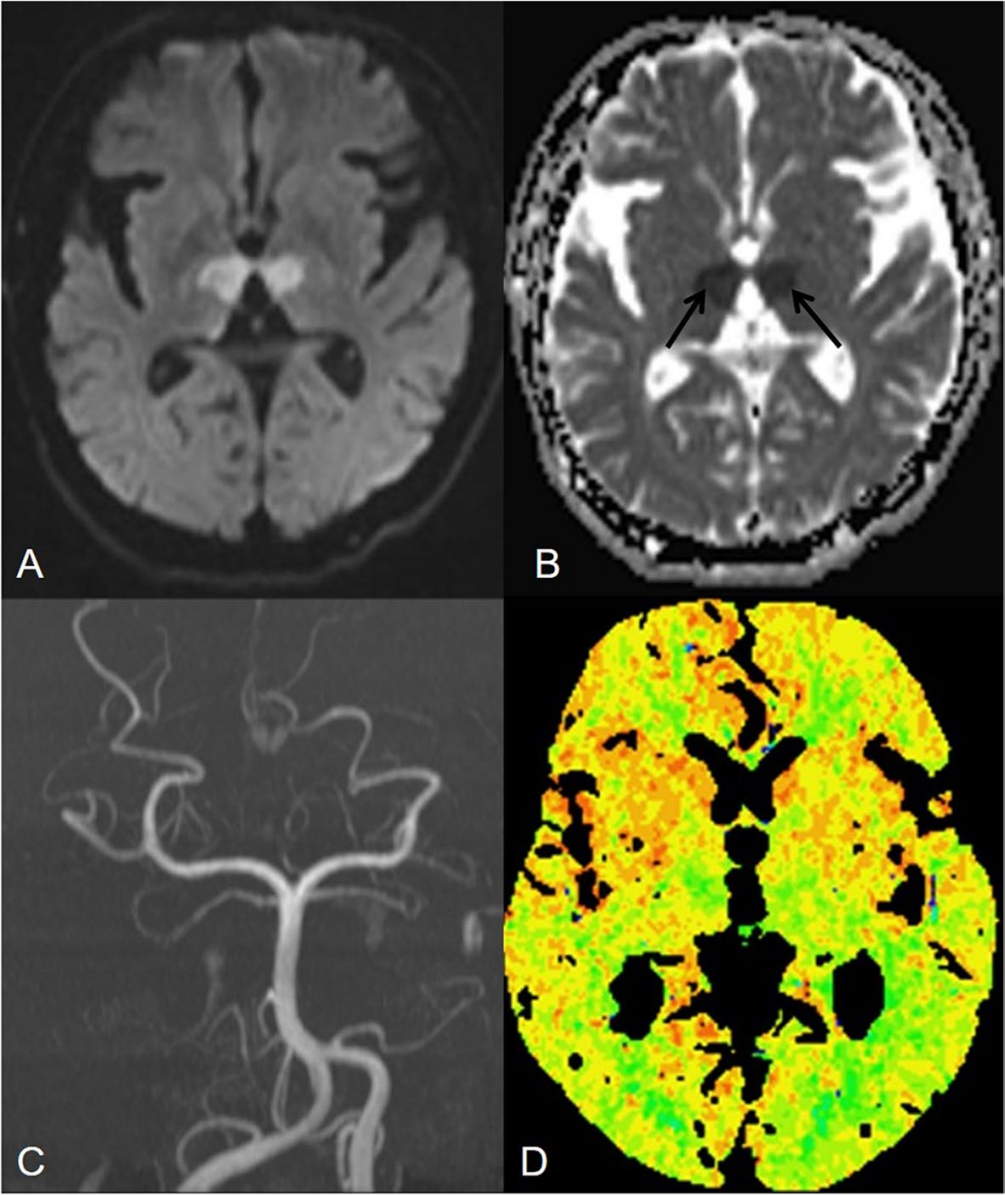
Acute symmetric infarctions in the bilateral paramedian thalamus on MRA and CT perfusion. Case 23. Axial DWI and ADC map (**A** and **B**) show acute symmetric infarctions in the bilateral paramedian thalamus (black arrows). MRA demonstrate no occlusion or stenosis in the posterior circulation (**C**). Hyperacute CT perfusion imaging with white arrows identifying areas of decreased mean blood flow (**D**)

Head CT of 22 patients with acute AOP infarctions performed 2 hours after the onset of symptoms showed no acute hemorrhage or ischemia, only one patient had bilateral parathalamic and rostral midbrain hypodensity (Fig. 3). One patient had bilateral paramedian thalamus and left anterior thalamus hypodensity at 24 hours after onset (Fig. 4). MR imaging showed characteristic asymmetricall bilateral paramedian thalamic in 30% (7/23) and symmetrical in 22% (5/23). Acute infarctions of paramedian thalamic and the rostral midbrain was present in 30% of patients (7/23), a distinct pattern of V-shaped hyperintensity on axial DWI along the pial surface of the midbrain in the interpeduncular fossa (Fig. 3) was found in 17% cases (4/23). Bilateral paramedian thalamic ischemia with anterolateral paramedian territory (Fig. 4) in 13% of patients (3/23). Bilateral paramedian thalamic infarction with left red nucleus (Fig. 5) in 4% of patient (1/23). None of the cases had a paramedian bithalamus and anterior thalamic with rostral midbrain. Stenosis or occlusion of the vertebral artery, basilar artery or P1-segments of PCA was observed in 5 patitens, whereas in 15 patients MRA was normal. CT perfusion image demonstrated symmetrically increased mean transit time (MTT) and decreased mean cerebral blood flow (CBF) and mean cerebral blood volume (CBV) in the bilateral paramedian thalami in the in 4 patients (Fig. 6). No bleeding signal was found in SWI of 4 patients.

### Treatment

All patients were classified as ischemic cerebrovascular disease after admission treatment. Therapeutic schedule included antiplatelet/anticoagulation, plaque stabilization and collateral circulation improvement. Patient *No*.4/8/11/14/16/19 were given alteplase in the time window (time window<4.5 h to 6 h). Heparin has also been used post-recanalization after thrombolysis. Low-molecular-weight heparins calcium injection (4000 IU/12 h) by subcutaneous was given to patients No.5/7/9/12/13/17 due to cardioembolism. Patients No.1/2/6/15 were given aspirin 200 mg/d orally. Patients No.3/10/18/20/21/22/23 were treated with aspirin 200 mg/d combined with clopidogrel 7.5 mg/d.

All patients were followed up for 3 months upon discharge. MRS score was shown in Table 1. There were 65% cases of a favorable outcome (MRS score ≤ 2) who experienced a good functional recovery and could carry out daily life activities. Patients partially recovered normal mental status. Patient *No*.4 was full patient recovery. Patient *No*.1/2/6/10/13/15/18/19/20 of vertical ophthalmoplegia recovered in various degree. Patient *No*.3/10/19/23 had memory loss and patient *No*.5/11/19 had obvious recent memory decline. Patient *No*.7/8/16/22 had unclear articulation. Patient *No*.2/6/8/9/10/13/17/18/20/22 had dyskinesia. The patients with rostral midbrain infarction were dependent and unable to walk without assistance. Patient *No*.5/7/9/10/13/18/20 still had disorientation or mild ataxia symptoms.

## Discussion

### Etiology and risk factors

Acute ischemia of AOP is uncommon, which accounted for 0.1–0.3% of all cerebral infarction [13, 14]. However, this is likely a conservative estimate due to the insufficient knowledge of it and limitations in the inclusion criteria [15]. AOP arising from P1 segments of the PCA supply predominantly paramedian bithalami with variable contribution to the rostral midbrain or anterolateral thalami [16]. In our studies, hypertension, smoking, diabetes mellitus, and hyperlipemia are risk factors for acute percheron infarction. Small vessel disease and arteriosclerosis are the causes of acute AOP infarcts in 14 patients (61%), which is similar to the previous reports [17]. It is indicated that cardioembolism is the most common cause [18], while only 7 cases (30%) had a potential source of cardiac embolism in our series. In addition, basilar artery dissection is seen in patient *No.*21 and 22. Atherosclerosis and small vessel disease are the main causes of acute AOP infarction.

### Relationship imaging diagnosis and clinical features

Acute AOP ischemia has great variability with respect to symmetry, size, and territory, which is mainly due to the thalamic arteries vary between individuals. These differences are related to the parent vessel from which each branch arises, the number and position of the arteries and their tributaries [9, 12, 18]. Paramedian bithalami of the 23 patients showed high signal intensity on FLAIR with restricted diffusion and hypointense in the ADC map (positive in 100%). Valentina Francioni et al. [19] presented mismatch between DWI and FLAIR mismatch of hyperacute paramedian bithalami ischemia, which points out the DWI and ADC map is very important for the differentiation and therapy in acute AOP infarction. Although head CT is not sensitive to acute AOP infarction, it is indispensable to exclude cerebral hemorrhage at admission. It is identified a case describing AOP ischemic on acute CT perfusion imaging for the first time, however, thrombolysis was not performed in that symmetrical perfusion abnormalities were not recognized [20]. Most interestingly, we found that patient No.4/8/11/14 show corresponding bithalamic ischemia changes on acute CT perfusion imaging and was given thrombolysis to avoid deterioration. DSA rarely shows the presence of AOP, more often than not, the AOP is too small to be visualized by angiography. Similarly, although the AOP is rarely visualized with conventional MRA, it is very valuable to observe the stenosis of BA and PCA by MRA.

Obstruction of the AOP oftentimes leads to ischemia of the midbrain (57%) and/or the anterior thalamus (19%) [21], while we found that acute infarction of the rostral midbrain was present in 30% and anterior thalamus in 13%. The polar artery is absent in 30–60% of the population in that PcomA is highly variable (absent or hypoplastic) [22]. Then, paramedian arteries not only may supply the paramedian but also the anterior thalamic territories [11, 23], especially when the polar artery is absent (Fig. 2). Therefore, an infarction of the paramedian bithalamus and the anterior thalamus may be explained by occlusion of a single AOP, rather than synchronous occlusions of an AOP and the polar artery. In addition, patient *No*.7 showed bilateral paramedian thalamic infarction with only left red nucleus.

Similar to previous studies [23, 24], we also analysis the relationship between AOP infarction region and clinical presentation. Based on previous report,^6, 9^ No case involving bilateral paramedian and anterior thalamic infarction with midbrain involvement was found. The thalami contain reticular and intralaminar nuclei, associative nuclei, effector nuclei, sensory nuclei and limbic nuclei. Acute infarction of AOP destroys these nuclei in different combinations and result in complex syndromes depending on which nuclei is involved [8]. The typical clinical symptoms are “altered mental status, vertical gaze palsy, and memory impairment”. Other symptoms include: disorientation, hemiplegia, cerebellar ataxia, movement disorders and dysarthria [25]. Disorder of consciousness in 22 cases, except patient No.15, may be due to involvement in the reticular activating system. Excitedly, recent evidences suggest that the paraventricular nuclei supplied the AOP is a key wakefulness-controlling nucleus in the thalamus [26]. The vertical gaze palsy observed in patient No.2/10/13/18/20 with BPTRMI is due to an associated involvement of the midbrain tegmentum, including the interstitial nucleus of Cajal and rostral interstitial nucleus medial longitudinal fasciculus (RINMLF). Meanwhile, vertical gaze palsy in patient No.1/15/19 with BPTI were also reported. The reason may be that the thalami have a vital role in cortical input processing to the RINMLF [17]. Memory dysfunctions are reported on patient No.3/5/9/10/11/19/23 (BPATI in patient No.5/11/19). The dorsomedial nucleus located in paramedian territory is supplied by the AOP, the mammillothalamic tract and anterior nucleus located in anterior territory is supplied by polar artery or AOP, which is usually related to memory function [27]. When the anterior thalamic territory is also involved, memory impairment is typically more severe [8]. In our series, patient No.5/11/19 with BPATI still have memory deficit after 90 days. Oculomotor nerve palsy including ptosis, ocular movement disorders and ocular abnormal reflexes in patient No.2/6/10/13/18/20 with BPTRMI is related to acute ischemia of the oculomotor nucleus located in near midline of mesencephalic. The mesencephalothalamic or thalamopeduncular syndrome included movement disorders, hemiplegia and cerebellar ataxia occurs in patients with BPTRMI is related to midbrain involvement [28]. The superior mesencephalic or rubral artery branch separately from P1 segment of the PCA or share a common origin with AOP, which supply blood to the interpeduncular nucleus, medial part of the red nucleus, nucleus of cranial nerve III and anterior part of the periaqueductal gray matter located in dorsal midbrain [29]. Similar to polar artery, a single AOP may result in BPTRMI, rather than synchronous occlusions of an AOP and the superior mesencephalic or rubral artery.

### Treatment and prognosis

**Fig. 7:**
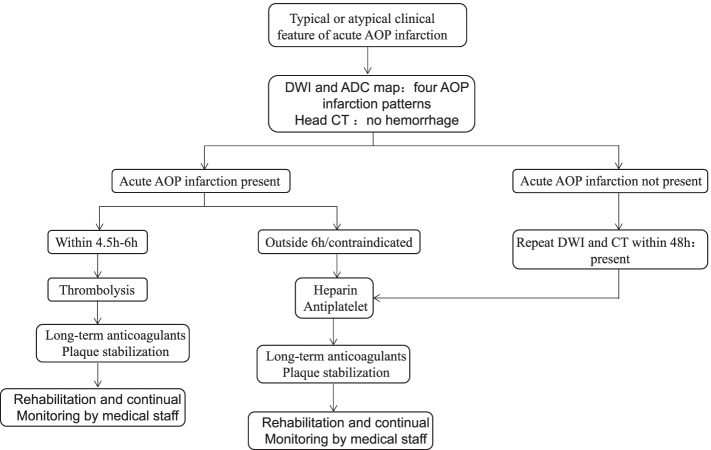
Algorithm for the treatment of artery of Percheron (AOP) infarction

In this study, 65% of the patients have a favorable outcome. Complete recovery from bilateral paramedian thalamic infarction has occasionally been reported [8]. At present, the goal of acute AOP occlusion treatment is to promote recanalization [30]. Patient *No*.4/8/11/14/16/19 were treated with thrombolytic therapy without cerebral hemorrhage or edema on head CT within 6 hours. Patient *No*.4 recovered completely and the residual cognitive impairment disappeared (MRS score = 0). Patient *No*.8/11/14/16/19 were fully conscious with partial memory deficit (MRS score = 1). Thrombolytic therapy (time window < 4.5 h–6 h) is considered to be the most effective treatment for acute AOP infarction [31]. Our results are consistent with previous studies. Patients who are not suitable for thrombolysis beyond 6 hours received antiplatelet and heparin therapy. We found a favorable outcome (MRS score = 1 or 2, except patient No.7) in patient No.1/3/5/12/15/17/21/22/23 with BPTI. Meanwhile, an unfavorable outcome (MRS score = 3 or 4) was present in patient No.2/6/9/13/18/20 with BPTRMI, which is consistent with previous studies [10]. All 23 patients we reported took oral anticoagulants to prevent recurrence. According to the literature reports and our cases, we summarized the flow chart of the algorithm for the treatment of acute AOP infarction (Fig. 7).

### Differential imaging

**Fig. 8:**
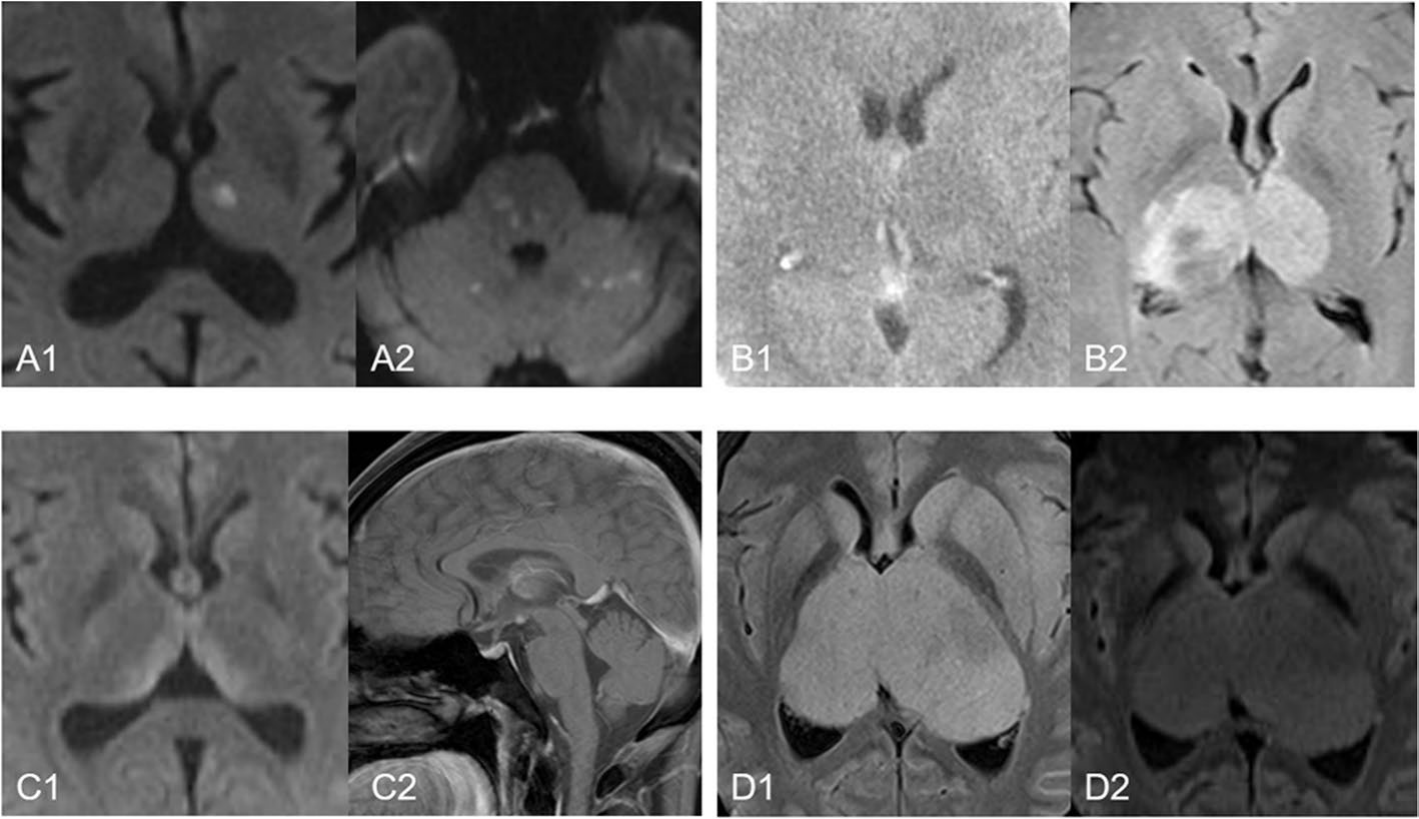
Differential diagnosis of AOP infarction. Top of the basilar artery syndrome can lead not only to thalamic infarction, but also to additional characteristic cerebellar, brainstem and occipital lobe infarction (**A1** and **A2**). For deep vein infarction caused by thrombosis, high density of bilateral internal cerebral veins (**B1**, black arrow) can be observed on head CT, and FLAIR shows angiogenic edema involving multiple arterial areas of thalamus (**B2**). Wernicke’s encephalopathy often implicates pulvinar (**C1**) with abnormal enhancement of papillary body (**C2**, white arrow). Diffuse midline glioma in bilateral thalamus with mass effect is hyperintense on FLAIR and isointensity on DWI (**D1** and **D2**)

There are some other etiologies to consider, such as the “top of the basilar” syndrome, deep venous infarction by thrombosis, wernicke’s encephalopathy and diffuse midline gliomas [32], which should not be confused with AOP infarction (Fig. 8). A single large embolus at the basilar artery tip could mimic acute AOP infarction pattern, but it often accompanies with additional characteristic cerebellar, brainstem and occipital lobe infarcts. Deep venous infarction caused by thrombosis and diffuse midline gliomas do not involve the specific blood-supply territory of the AOP, but multiple arterial regions. Wernicke’s encephalopathy affects not only the thalamus but also the mammillary bodies, tectal plate, and periaqueductal area. Diffuse midline gliomas in bilateral thalamus with space-occupying effect are hypointense on T1WI and hyperintense on T2WI or FLAIR as well as isointensity on DWI.

### Limitation

There are some limitations in thist study. Firstly, all the 23 patients were retrospectively enrolled from a single institution. Due to the limited sample size, large scale multicenter research is necessary in the future. Secondly, although many researchers believe that thrombolytic therapy is the first treatment option for acute AOP infarction, there are few cases of AOP occlusion recorded in this study. In our study, only 6 patients received thrombolytic therapy. The development of treatment protocols needs to be further improved through systematic case reports and clinical trials.

## Conclusion

AOP is a rare anatomical variation of arteries. Due to the small lumen of AOP, angiography usually cannot show occlusion of AOP. Therefore, angiography should not be used in the diagnosis of acute AOP infarction. DWI and ADC are the best methods to diagnose acute AOP infarction. Clarifing the relationship between imaging and clinical manifestations is helpful to improve the disease management of acute AOP infarction.

## Data Availability

The datasets generated and/or analysed during the current study are not publicly available due to limitations of ethical approval involving the patient data and anonymity but are available from the corresponding author on reasonable request.

## Abbreviations

ADC: Apparent diffusion coefficient
AF: Arteriovenous fistula
CTP: Computed tomography perfusion
AOP: Artery of percheron
BPATI: Bilateral paramedian and anterior thalamic infarction
BPTI: Bilateral paramedian thalamic infarction
BPTRMI: Bilateral paramedian thalamic with rostral midbrain infarction
BPTRNI: Bilateral paramedian thalamic with red nuclei infarction
CT: Computed tomography
DSA: Digital subtraction angiography
MRA: Magnetic resonance angiography
MRI: Magnetic resonance imaging
MRS: Modified Rankin scale
PCA: Posterior cerebral artery
SWI: Susceptibility weighted imaging
